# Point-of-Care Testing for Pharyngitis in the Pharmacy

**DOI:** 10.3390/antibiotics9110743

**Published:** 2020-10-28

**Authors:** Sabiha Essack, John Bell, Douglas Burgoyne, Wirat Tongrod, Martin Duerden, Aurelio Sessa, Attila Altiner, Adrian Shephard

**Affiliations:** 1 Antimicrobial Research Unit, College of Health Sciences, University of KwaZulu-Natal, Durban 4041, South Africa; 2 Graduate School of Health, University of Technology Sydney, Ultimo, NSW 2007, Australia; john.bell@nexonline.com.au; 3 College of Pharmacy, University of Utah, Salt Lake City, UT 84112, USA; burgoyned@gmail.com; 4 Faculty of Pharmaceutical Sciences, Huachiew Chalermprakiet University, Samut Prakan 10540, Thailand; freshwirat@yahoo.com; 5 School of Medicine, Centre for Medical Education, Cardiff University, Cardiff CF14 4XN, UK; martin@theduerdens.co.uk; 6 Italian College of General Practitioners and Primary Care (SIMG, Società Italiana di Medicina Generale delle Cure Primarie), 50142 Florence, Italy; sessa.aurelio@simg.it; 7 Institute of General Practice, Rostock University Medical Center, 18055 Rostock, Germany; altiner@med.uni-rostock.de; 8 Reckitt Benckiser Healthcare Ltd., Slough SL1 3UH, UK; Adrian.Shephard@rb.com

**Keywords:** antimicrobial stewardship, pharyngitis, upper-respiratory tract infections, viral infections, bacterial infections, point-of-care testing, pharmacy, antimicrobial resistance, streptococcal infections, antibiotics

## Abstract

Pharyngitis (also known as sore throat) is a common, predominately viral, self-limiting condition which can be symptomatically managed without antibiotic treatment. Inappropriate antibiotic use for pharyngitis contributes to the development and spread of antibiotic resistance. However, a small proportion of sore throats caused by group A streptococcal (GAS) infection may benefit from the provision of antibiotics. Establishing the cause of infection is therefore an important step in effective antibiotic stewardship. Point-of-care (POC) tests, where results are available within minutes, can distinguish between viral and GAS pharyngitis and can therefore guide treatment in primary healthcare settings such as community pharmacies, which are often the first point of contact with the healthcare system. In this opinion article, the evidence for the use of POC testing in the community pharmacy has been discussed. Evidence suggests that pharmacy POC testing can promote appropriate antibiotic use and reduce the need for general practitioner consultations. Challenges to implementation include cost, training and ‘who prescribes’, with country and regional differences presenting a particular issue. Despite these challenges, POC testing for pharyngitis has become widely available in pharmacies in some countries and may represent a strategy to contain antibiotic resistance and contribute to antimicrobial stewardship.

## 1. Introduction

Pharyngitis, also known as sore throat, is one of the most common reasons for which patients present to their general practitioner (GP) [1]. Though pharyngitis is usually an acute, non-serious condition, the symptoms can be painful and may have a significant impact on a patient’s quality of life [2]. The desire to obtain relief from painful symptoms of sore throat is a major driving factor in patient consultations with healthcare professionals (HCPs) in primary care and of antibiotic-seeking behavior [3,4].

The majority of respiratory tract infections (RTIs), including pharyngitis, are caused by viral infections [5]. Pharyngitis is of bacterial etiology in approximately 5–30% of cases, with approximately 5–10% of these cases attributed to group A streptococcal (GAS) infection in adults [5,6,7]. Pharyngitis is generally a self-limiting condition regardless of whether the cause is bacterial or viral, and approximately 90% of people recover without any treatment within 1 week [8]. Nevertheless, antibiotic misuse (both overuse and inappropriate choice of antibiotics) for pharyngitis remains commonplace in primary care, with many clinicians not following current guidelines on GAS pharyngitis [9,10]. Approximately 60% of sore throat consultations result in an antibiotic prescription [11], despite the fact that antibiotics are ineffective against viruses and are therefore inappropriate for up to 90% of pharyngitis cases [5,8,12,13].

Inappropriate use of antibiotics for pharyngitis not only increases the risk of unnecessary side effects for patients [12,14], but results in a higher risk of antibiotic resistance developing and spreading within communities [15,16,17,18,19]. However, in cases of GAS pharyngitis, timely initiation of antibiotics is usually recommended to prevent the spread of infection to close contacts, modestly reduce the duration of symptoms, and limit rare long-term complications [8,20]. As such, careful antibiotic stewardship is required for conditions such as pharyngitis, in which overuse and misuse of antibiotics is common [9,10,11]. Patients with pharyngitis presenting to primary care services may in fact be looking to understand the cause of their sore throat and obtain pain relief, rather than acquire antibiotics [3]. An important step in effective antibiotic stewardship is therefore to establish whether there may be an indication for antibiotics by ascertaining a non-viral etiology. However, distinction between bacterial and viral pharyngitis by clinical observation alone is often challenging, and conventional diagnostic methods, such as throat culture tests, have lengthy turnaround times [20,21,22]. Point-of-care (POC) tests, in which the results are available in minutes, are thus very valuable in primary healthcare settings such as community pharmacies, which are often the first point of contact with the healthcare system in both high- and low-income countries [23,24].

As a consequence of the ongoing coronavirus (COVID-19) pandemic, throat swab testing has become commonplace in the community setting. As of 13 July 2020, an estimated 230,832,017 tests for COVID-19 have been carried out in the 30 most impacted countries worldwide [25]. The full impact of community throat swab testing for COVID-19 has not yet been established. However, test results are being used to support appropriate clinical management of patients, identify infected individuals in order to contain the disease, inform policy decisions for government strategies and monitor the circulation of the virus in the community [26].

It is not yet clear whether the current widespread use of throat swabs as a result of the COVID-19 pandemic will have implications for the use of POC testing in the community for other conditions, such as pharyngitis, in the future. However, the increased patient experience in receiving and in some countries self-administering throat swabs and the mechanisms put in place to facilitate these testing programs could mean that POC testing becomes more commonplace following the pandemic. Nonetheless, pharmacists are well placed to carry out such tests, and POC testing for pharyngitis is already available in pharmacies in some countries [27]. The aim of this paper is to discuss the use of POC testing for GAS pharyngitis in the community pharmacy setting to help determine whether antibiotics may be warranted and to ensure their appropriate use.

## 2. Inaccuracy of Clinical Assessment for Bacterial Sore Throat

Accurate diagnosis of GAS pharyngitis using clinical symptoms alone is generally difficult due to overlap of clinical signs and symptoms between bacterial and viral infections [20]. The Centor and McIsaac criteria are examples of clinical scores that can be used to diagnose GAS pharyngitis. The Centor criteria in particular has been endorsed by clinical guidelines as a means to inform clinical decision making for management of pharyngitis in adults. For example, the American College of Physicians (ACP) recommend that patients with a Centor score of ≤1 should not receive antibiotics or further testing for GAS pharyngitis, those with a score of 2 or 3 should undergo rapid Streptococcus testing and receive antibiotic treatment if positive, though patients scoring 3 or ≥4 can be managed with empirical antibiotic therapy [28]. However, the accuracy of using clinical assessment to assess GAS infection has been questioned in several studies. A 2020 meta-analysis of diagnostic test accuracy studies conducted in primary care settings showed that the Centor and McIsaac scores only provide ‘fair discrimination’ of those with and without GAS. The study concluded that alternative methods, such as POC tests, may be required to definitively diagnose GAS pharyngitis. [29].

A study by Shephard et al. (2015) demonstrated that the sensitivity of clinical assessment (CAST) is low (27.5%), as is the specificity (79.7%). The predictive accuracy of CAST was also low; 86.9% of patients would have taken antibiotics unnecessarily if diagnosis of GAS was based on clinical features alone, and 9.2% of patients with culture-proven GAS would have not been treated with antibiotics [30]. Similarly, Orda et al. (2016) evaluated the diagnostic accuracy of clinical decision making in pediatric patients presenting to an emergency department in Australia. The study found that the positive predictive value of clinician decision making for a positive GAS swab was 29% (95% Cl: 17–43), meaning that 71% of patients considered to have GAS based on clinical assessment would have been prescribed antibiotics inappropriately [31]. Moreover, the negative predictive value was 78% (95% CI: 63–88), indicating that 22% of patients with culture- proven GAS would not have received antibiotics if diagnosis was based only on clinical assessment.

Traditional culture methods may also present a potential delay in treatment due to turnaround times, which can take up to 18–48 h [22]. In contrast, POC testing for GAS gives results within minutes, with high sensitivity compared to testing with throat swabs [32]. POC tests are carried out using a sterile throat swab that is rubbed over the tonsillar area and posterior pharynx, taking care to avoid contamination by not touching other areas of the throat and mouth [20]. A wide variety of rapid tests are commercially available [33]. Rapid antigen detection tests (RADTs) use immunoassay detection methods, such as immunochromatography or immunofluorescence, to detect the presence of Strep A antigens. The time to result for these rests is typically approximately 5 min, excluding the rest of the consultation and sample preparation time. Depending on the manufacturer, results can be read using visual inspection or by using a test reading device. Some rapid tests implement molecular methods of detection, such as polymerase chain reaction (PCR) and isothermal nucleic acid amplification, though primary care settings such as the pharmacy are less likely to have this kind of technology on site. For molecular tests, time to result is slightly longer than with RADTs, with read times ranging from <8 to ≥18 min depending on the type of test used [33].

In the aforementioned study by Shephard et al. (2015), the sensitivity and specificity of rapid POC tests for GAS pharyngitis was estimated to be 87.5% and 96.8%, respectively [30]. Moreover, Lean et al. (2014) conducted a meta-analysis of data from 48 studies looking at the diagnostic accuracy of POC testing for GAS in adult and pediatric patients. Compared to standard throat culture swabs, pooled estimates for the sensitivity and specificity of POC testing were 86% (95% CI: 0.83–0.88) and 96% (95% CI: 0.94–0.97), respectively, with similar estimates recorded for pediatric patients [34]. Another study by Stewart et al. (2014) found that POC immunochromatographic methods show high specificity (93%) and sensitivity (91%) when diagnosing GAS pharyngitis in adult populations, but not in children [35]. However, like traditional culture testing methods, POC testing interventions cannot distinguish between GAS pharyngitis and asymptomatic GAS carriers with viral pharyngitis [36]. In high-income countries, the rate of asymptomatic carriage is approximately 7.5%. This should be considered in active POC testing programs, as the benefits of antibiotic treatment for patients who test positive for GAS may be limited in populations where the prevalence of GAS is similar to the asymptomatic carriage rate. In these situations, the positive GAS test could detect incidental GAS which may not be causative of the patient’s symptoms [37].

## 3. Effect of POC Testing on Appropriate Antibiotic Prescribing in Primary Care

The use of POC testing could improve antibiotic use and reduce patient pressure for antibiotic prescriptions [38]. For example, a retrospective analysis by Luo et al. (2019) showed that antibiotic usage was less frequent in patients who were tested for GAS using certain POC tests, such as nucleic acid amplification testing, compared to those who received no test [9]. Multiple studies have investigated the impact of POC testing on antibiotic prescribing in primary care, in both general practice and community-based care.

## 4. POC Testing in General Practice

A number of studies have focused on the use of POC testing in a physician setting. Worrall et al. (2007) carried out a randomized, controlled trial comparing methods to diagnose GAS pharyngitis in urban and suburban family practice offices in Canada. The study found that antibiotic prescription rates were significantly lower in patients who had a GAS POC test alone or combined with sore throat decision rules, compared to those diagnosed according to usual clinical practice (26.7%, 38.2% and 58.2%, respectively, *p* < 0.001) [13]. The efficacy of POC testing in reducing inappropriate antibiotic use has also been demonstrated in pediatric populations. Bird et al. (2018) observed similar results in a study that assessed antibiotic prescribing rates before and after introducing the McIsaac clinical scoring tool and GAS POC testing in a pediatric emergency department in the United Kingdom (UK). Antibiotic prescribing rates decreased from 79% at baseline to 24% in the first year and 28% in the second year after combined use of POC testing and the McIsaac clinical scoring tool [39]. In a prospective, open-label study, Rao et al. (2019) investigated the impact of POC testing in pediatric patients with pharyngitis presenting to a large pediatric clinic in the United States of America (USA). The findings of the study indicated that antibiotics were appropriately prescribed in 87.5–97.1% of cases, depending on the POC testing method used [40].

In contrast to the aforementioned studies, Little et al. (2013) reported that use of antibiotics was comparable between patients attending GPs in the UK who were randomized to receive a POC test for GAS after receiving a high clinical score (the FeverPAIN score) and those who were diagnosed according to clinical score alone (35% and 37% respectively, compared with 46% in patients who received a delayed prescription for antibiotics). The rate of immediate prescriptions for antibiotics was also comparable between groups, though fewer delayed prescriptions were administered with the POC test compared to the clinical scoring group. The study concluded that compared to using clinical score alone, there was no evidence to justify the increased time and cost of using POC testing. The authors suggested that this limited additional value could in part be due to the fact that the ability of the test to identify GAS is matched by its inability to diagnose group C and G streptococcal infections, which cause similar symptoms to GAS. It is possible that differences in the type of clinical score used may also account for discrepancies between studies [41].

The question also stands as to whether physicians will change their antibiotic-prescribing behaviors in response to the availability of POC testing. In some cases, doctors may prescribe antibiotics regardless of a positive test result [42]. For example, in a qualitative study on GP perceptions of the introduction of POC testing for common infections into routine primary care, Butler et al. (2008) highlighted that practitioners may be concerned about the reliability of POC tests and be unsure on whether to prescribe antibiotics in these situations [43].

## 5. POC Testing in the Pharmacy Setting

Several community-based pharmacy studies have demonstrated success using POC testing to reduce inappropriate antibiotic prescribing. Demoré et al. (2018) conducted a community pharmacy-based antimicrobial stewardship intervention in France which offered free POC testing to adults with sore throat. According to POC testing, 8.3% of patients were positive for GAS, all of whom further consulted a physician and were prescribed an antibiotic treatment. This was in contrast to patients with negative test results, of whom 96.5% did not seek further consultation. Those who received a diagnosis of viral pharyngitis received educational materials and advice about appropriate symptomatic relief [44]. A similar study by Papastergiou et al. (2018) implemented community pharmacist-directed GAS POC testing in Canada and found that 25.5% of patients tested positive for GAS infection. Antibiotics were administered within the same day in 68.7% of positive cases. Despite being required to pay $20 for the test, 82% of patients indicated that they were ‘very likely’ to use the service again if needed, and 72% were ‘very satisfied’ with the sore throat screening test [45].

In addition to reducing inappropriate antibiotic prescribing, community-based POC testing could improve access to care for patients. Klepser et al. (2016) reported that 17.6% of patients were positive for GAS pharyngitis in a community pharmacy-based POC testing study in the USA, and approximately 16.8% of these patients received antibiotics. In cases where the patient tested negative for GAS, the pharmacist recommended appropriate over-the-counter (OTC) symptomatic relief products and discussed the diagnoses with the patient. Of the patients who attended the testing service, 43.9% attended the pharmacy outside of normal surgery opening hours and 43.2% had no primary care provider. The service was priced at $75, although all eligible patients were provided with a voucher to cover this cost [46]. Likewise, a retrospective study by Klepser et al. (2018) investigated whether pharmacy-based community testing for GAS pharyngitis and influenza in the USA could improve patient care, reporting that 16.9% of sore throat patients were positive for GAS, of whom 98.9% received antibiotics. Treatment with OTC symptomatic relief products was recommended for 99.8% of the patients who tested negative for GAS. Of the patients tested for GAS pharyngitis and influenza, 38% presented to the pharmacy outside of normal clinic hours and 53.7% did not have a primary care provider [47].

Mantzourani et al. (2020) studied the impact of a National Health Service (NHS)-funded POC sore throat test and treat (STTT) service in selected pharmacies in Wales (UK) on antibiotic use, patient safety and GP consultation rates. In total, less than 20% of the 1725 consultations resulted in antibiotic supply. GP consultation rates were found to be lower than the equivalent monthly average, and a total of 93% of patients would have consulted their GP if the service had not been available [48]. Likewise, in a study on a patient-funded POC STTT in 35 community pharmacies in England, only 9.8% of sore throat consultations resulted in the prescription of antibiotics. Of the patients who were not exhibiting signs of a bacterial infection (based on a score of 1 or 2 on the Centor scoring system) 48.8% of patients would have consulted their GP if the testing service had not been available [49].

## 6. POC Testing and COVID-19

Careful antibiotic stewardship is of heightened importance in the current landscape, with treatment of patients with COVID-19 and suspected secondary bacterial infection potentially increasing the risk of antibiotic resistance [50]. Though there is a lack of data on the management of non-hospitalized COVID-19 cases and antibiotic use, the inappropriate use of antibiotics for viral infections, such as upper-respiratory tract infections (URTIs), has been extensively documented at a community level. Thus, diagnostic stewardship and appropriate antibiotic prescribing should be exercised when treating COVID-19 in order not to escalate the pre-existing risk of antibiotic resistance [50]. A recent review by Rawson et al. (2020) suggests that broadening the roles and responsibilities of HCPs and the development of rapid diagnostic tests to support prescribing decisions are possible interventions to tackle the increased rates of antibiotic prescribing for patients presenting with respiratory symptoms during the COVID-19 pandemic [51]. Though these interventions would initially be geared specifically towards patients with COVID-19, it is possible to speculate that the SARS-CoV-2 virus may prove to be a catalyst for change in diagnostic practice on a wider scale in the future. Such changes are already evident in some countries, where governments have granted legal extensions to the role of pharmacists in light of the COVID-19 pandemic [52]. In Florida, legal extensions granting pharmacists permission to not only screen for COVID-19, but to test for and initiate treatment of influenza and GAS infection represent a pertinent example of how the COVID-19 pandemic could facilitate change to diagnostic practices for conditions such as pharyngitis [52].

## 7. Pharmacist’s Contribution to Antimicrobial Stewardship and to Deliver POC Testing

For antibiotic stewardship to be effective, collaboration between prescribers, pharmacists and patients is essential [53]. Community pharmacists are at the forefront of primary care and are ideally placed as antibiotic guardians, possessing the knowledge, opportunity and commitment that is key for effective antibiotic stewardship. Community pharmacists possess a specialist knowledge of medicines that means they have the capability to advise on prudent antibiotic use. For many patients, community pharmacists are the most accessible healthcare providers and owing to the contact pharmacists have with both patients and prescribers, there are multiple opportunities for antibiotic stewardship within the pharmacy setting [53]. It should be emphasized that antibiotic stewardship may take on different forms depending on geographical context. For some people living in certain parts of the world, such as Australian Aborigines for whom acute rheumatic fever is common, antibiotics may be justified to reduce the risk of serious complications from GAS infection [8]. In other parts of the world rheumatic fever is incredibly rare, and the risk of complications from GAS infection may be similar to the risks arising from inappropriate use of antibiotics (such as allergic reactions) [8,20,54].

**Figure 1:**
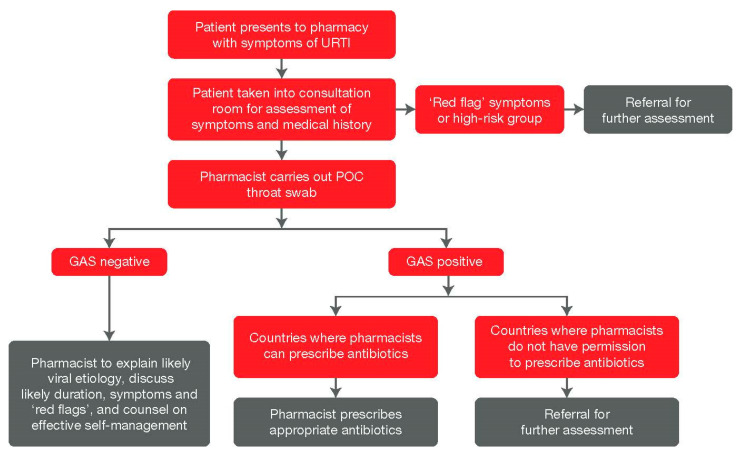
Proposed screening process and methodology for POC testing in the community pharmacy. GAS: group A Streptococcus; POC: point-of-care; URTI: upper-respiratory tract infection.

Guidelines advocate symptomatic management as a first-line treatment for pharyngitis [54,55]. Pharmacists have a vast knowledge of both prescription and OTC medications, which makes them ideally placed to advise on evidence-based OTC symptomatic relief products and advise patients when a referral to a doctor is necessary [53]. The implementation of POC testing for pharyngitis in the community pharmacy could help pharmacists determine those who would benefit from evidence-based symptomatic management and those who require antibiotic treatment or a doctors referral [44,46,47]. Figure 1 details a proposed process for how POC testing could be conducted in the pharmacy:

Indeed, pharmacists can deliver a wide range of POC tests, including tests for pharyngitis [56]. Dulaney et al. (2018) carried out a questionnaire-based study in the USA, looking at pharmacists’ perceptions of POC testing and treatment for influenza and GAS pharyngitis in a community pharmacy setting. The results showed that 69% of pharmacists either strongly agreed or agreed that they would be willing to perform POC testing in a community pharmacy setting, and 86% either strongly agreed or agreed to be willing to recommend appropriate treatment for influenza and streptococcal pharyngitis. The majority of pharmacists (79%) either strongly agreed or agreed that they had sufficient clinical knowledge to treat these infections, though it should be noted that 66% of pharmacists strongly agreed/agreed there were barriers to implementing POC testing services for pharyngitis in the community pharmacy setting [57]. Specific barriers identified by pharmacists included lack of reimbursement, training, resources and awareness of the service, as well as constraints relating to the pharmacy infrastructure. A similar study by Mantzourani et al. (2019) investigated pharmacist perceptions of the aforementioned NHS-funded POC STTT in Wales (UK) and found that all participants were enthusiastic about providing the service. However, the participants noted that some pharmacists may not be as willing to expand their role and provide additional services, which may present a barrier if the STTT were to be implemented nationally [58].

POC testing in the community pharmacy setting can be cost saving from a public funding perspective. Lathia et al. (2018) conducted cost-minimization analyses of community-based POC testing for GAS pharyngitis in five Canadian provinces from the public payer perspective [59]. Estimations of total cost savings ranged from $1.3 million to $2.6 million per year across the five provinces, indicating that community POC testing for GAS pharyngitis in pharmacies may lead to cost savings in comparison to physician-based care within publicly funded healthcare systems. The International Pharmaceutical Federation (FIP) also recognize the potential economic benefits of the implementation of community pharmacy-based POC testing services. In their statement of policy on POC testing in pharmacies, the FIP states that the provision of POC testing services in the pharmacy would have benefits for both publicly funded and insurance-funded healthcare systems [60].

## 8. Implementation Considerations and Challenges for Pharmacies

How antibiotics are provided in cases where patients test positive for GAS pharyngitis in the pharmacy should be considered before implementation of POC testing services in the community. One question to be raised is whether pharmacists should have the authority to prescribe antibiotics in response to a positive test result, and if so, how this will be regulated. This may vary in different countries [61]. Set legal frameworks could be used to address this issue [62]. For instance, in a pilot scheme in the UK, selected pharmacies in London were permitted to carry out POC testing for sore throat and administer antibiotics to patients who tested positive for bacterial tonsillitis under pharmacy-based patient group directions (PGDs). PGDs set out specific instructions which allow authorized, registered HCPs to prescribe specified medications to a pre-defined group of patients without the need to see a doctor [62]. In addition to PGDs, approximately 4000 pharmacists in the UK have independent prescribing capability, which gives them permission to autonomously prescribe medications [63]. This may need to be taken into account if POC testing were to be implemented nationally in order to avoid situations where some patients are able to obtain antibiotics immediately from their pharmacist whilst others experience a delay in treatment due to being referred back to their GP. It should also be noted that antibiotics are available without prescription in some countries. Where antibiotics are provided without prescription, legally in OTC medications or otherwise, patient expectations could impinge as much on pharmacists as they do on doctors [64]. Policy issues in some countries may also present a barrier to the implementation of POC testing in the pharmacy. For example, pharmacists in Germany are not permitted to diagnose patients by law [65].

Alignment between GPs and pharmacists is also critical to the success of POC testing in the pharmacy. Before implementation of POC testing in the community pharmacy setting, local agreements and protocols need to be developed to ensure that there is mutually shared trust between GPs and pharmacists and to prevent situations where both parties are prescribing antibiotics. Pharmacists may feel undervalued by GPs [66], and GPs may lack confidence in extended pharmacy services [67]. Thus, protocols should detail the process by which referrals between pharmacists and GPs would be carried out, in order to maximize the relationship between GPs and pharmacists. Mantzourani et al. (2019) reported that good communication between GPs and pharmacists enabled the successful communication of the service objectives prior to implementation of a STTT service in Wales (UK) and helped GPs to feel confident in recommending the service to their patients [58].

The pharmacy environment can create a number of barriers for the implementation of POC testing for diagnosis of GAS pharyngitis. Implementation of POC testing can often be costly, time consuming and resource intensive, and sufficient staffing levels are required to run the service well. According to a study conducted by Corn et al. (2018) in the USA, the average time taken for pharmacists to complete the entire patient encounter (pre-screening and consultation, performance of the POC test and counselling on treatment after the test) was 25.3 ± 4.8 min. The average pharmacist participation time per consultation was 12.7 ± 3.0 min, which significantly decreased to 2.6 ± 1.1 min when pharmacist interns were included in the testing procedure [68]. Demore et al. (2018) reported that community pharmacists in France spent 6–15 min to perform the POC test. Pharmacists in this study were also required to attend a 2 h teaching session before carrying out the intervention. However, the POC testing service was received well, with 98.6% of pharmacists who gave feedback declaring to be ready to implement this intervention in daily practice, if endorsed and reimbursed [44]. Another key consideration is how exactly pharmacists will be reimbursed for this time. Should pharmacies charge patients to cover this cost, or will they be reimbursed by a third-party payer such as the government or an insurance company? Out-of-pocket payment and lack of reimbursement or funding has been noted by pharmacists as a key barrier to implementing extended pharmacy roles in a number of countries, including the UK [69,70].

The National Institute of Health and Care Excellence (NICE) does not recommend the routine adoption of GAS POC tests in clinical practice in England and Wales [33]. NICE concluded that the use of POC tests was unlikely to be a cost-effective use of NHS resources when added to clinical assessment by GPs. Such guidelines may seem counter-intuitive given that POC testing has been previously demonstrated to reduce antibiotic prescription rates. However, this analysis was partly based on evidence that most sore throats get better without treatment, regardless of their etiology [8]. Furthermore, the NICE guidelines did not analyze the cost effectiveness of GAS POC in pharmacies. It is possible to speculate that there may be differences in the utility of POC testing between GPs and pharmacies; for example, it may be challenging to perform the test and address specific patient needs within the time frame of a standard doctor’s appointment [33]. Moreover, GAS POC tests could prove to be more cost effective in pharmacies where POC testing is funded by patients rather than the government [49], as patients who test negative for GAS pharyngitis can be immediately directed towards non-prescription symptomatic relief and no GP consultation is required [46,47].

A deficit in pharmacist training presents a significant barrier to implementation of POC testing in community pharmacies, with specific concerns including good laboratory practice, test performance and interpretation, knowledge of test shortcomings and requirements for treatment, record keeping and disease reporting [27]. In an interview-based study by Mantzourani et al. (2019) in the UK [58], pharmacists expressed concerns regarding a lack of training for a STTT service in Wales (UK), in particular around the decision-making process for differential diagnoses, when to refer the patient to another HCP, and how to perform the test [58]. It is also worth noting that studies on POC testing in the community pharmacy setting have generally focused on trained pharmacists [44,46,47], and there is a paucity of data on the willingness and ability of pharmacy counter staff to carry out POC testing. It is therefore important to reflect on who would be carrying out the POC test if these interventions were to be implemented on a wider scale. Moreover, global differences in training for POC testing should be considered. In a 2017 review on the use of POC testing in low- and middle-income countries (LMICs), Kuupiel et al. (2017) described how training for POC diagnostics can be hindered by a lack of infrastructure, technologies and material for production of tests in LMICs [71].

In some cases, the pharmacy organization and infrastructure itself can raise issues. Community pharmacies will need to ensure that they have liability insurance if they will be carrying out roles outside of those covered under a typical professional liability policy [72]. In some small pharmacies, the question of space may be an issue, as a consultation room would be required to carry out the test procedure [73]. This designated area should not only provide privacy for the patient, but have suitable facilities for sample collection, test execution and safe disposal of clinical waste [73]. The infectious nature of body fluids should also be considered in a setting such as the community pharmacy, where cross-infection between patients attending the practice could occur. Learnings from the implementation of drive-through screening centers for COVID-19 could be pertinent here, where tests are carried out in the individual’s car and subsequently processed, reducing the risk of contamination between patients [74]. A similar approach could possibly be used for other POC tests, considering that drive-through pharmacy services have become increasingly recognized across the world after being first introduced in the USA in the 1990s [75,76].

Even where pharmacies are equipped to carry out POC testing, the uptake of the service is not always as expected. At a general practice in Melbourne (Australia) offering eye swabs for viral conjunctivitis, only two patients underwent POC eye testing over a 3week period. At the same center, the onsite pharmacist dispensed 10 prescriptions for chloromycetin eye drops per day [77]. In Italy, many community pharmacies offer POC testing for patients presenting with sore throat, although the uptake is low [78]. In a rapid interview of 12 pharmacies in a local health district in Northern Italy consisting of 50,000 patients and 40 GPs, POC testing was only performed 2–3 times a month during wintertime [78]. The cost of the test for the patient and time taken to perform the test for the pharmacist are likely contributors to the low uptake of POC testing in this region [78].

In summary, it is evident that POC testing in the pharmacy is not without challenges. It replaces differential diagnosis and clinical judgement by a doctor with POC testing and consultation with a pharmacist. The pharmacist may only refer patients with overt “red flags” to the doctor, as evident from studies where tests were only offered to patients who were suspected to have GAS pharyngitis based on Centor or FeverPain scoring criteria, [44,46,48] and may thus miss other clinical signs and symptoms requiring medical attention. Differences in pharmacy regulations, enforcement capacity and ethical pharmacy practice also play a role. Pharmacists are legally allowed to prescribe antibiotics in some countries, while most countries require antibiotics to be dispensed on prescription by a medical doctor. Without adequate enforcement capacity, regulators depend on the professional ethics of pharmacists to refer the patient, but there is always the risk that pharmacists will dispense antibiotics OTC based on POC results despite regulations. This calls for adequate enforcement capacity as well as collaborative practice between doctors and pharmacists.

## 9. Country Examples and Recent/Ongoing Initiatives

Funding and pharmacy practice models vary greatly between countries, which may significantly impact the success of POC testing implementation. In the USA, POC testing for GAS pharyngitis is recommended by clinical guidelines and is available in community pharmacies across the country [9,79]. Pharmacists in many states are able to apply for a Clinical Laboratory Improvement Amendments (CLIA) waiver, which allows them to carry out POC testing in the community pharmacy setting [27]. Pharmacists providing POC testing services in the USA are able to charge patients directly, or in some cases, a third-party insurance company is willing to pay for the test [80].

The implementation of POC testing may be more difficult in countries where healthcare is publicly funded. In contrast to the USA, clinical guidelines in the UK do not recommend the use of POC testing for GAS pharyngitis in primary care, and POC testing is not routinely provided in many general practices or the pharmacy setting, though rapid tests may be available in some community pharmacies [33,58]. Since patients can access most healthcare services for free on the NHS, it may be unlikely that patients would agree to undergo POC testing in the pharmacy unless the service was also available to them free of cost. Additionally, POC testing kits are often more costly for the payer than a course of generic antibiotics, which can often be purchased inexpensively [33,81]. However, this cost comparison could be different in countries where GPs prescribe non-generic, premium antibiotics.

Implementation of POC testing may also be more feasible in countries where pharmacists already have authority to prescribe antibiotics. In South Africa, pharmacists are authorized to prescribe medicines, including antibiotics, from the Standard Treatment Guidelines/Essential Medicines List for Primary Health Care upon completion of the Primary Care Drug Therapy (PCDT) course and registration with the South African Pharmacy Council [82,83,84]. Implementation of POC testing for GAS in South Africa to reduce inappropriate antibiotic use for pharyngitis could advance the antibiotic stewardship role of pharmacists who are already PCDT certified.

Acceptance of community pharmacists as clinicians also varies between countries and could impact the success of POC testing services. In countries such as the UK, Canada, USA and Australia, it is common for community pharmacists to carry out a variety of extended services [69,85]. The right to prescribe has also been extended to some community pharmacists in these countries, and acceptance of pharmacists as prescribers is growing [69,85]. However, in countries such as Pakistan, Singapore, Hong Kong, Sudan and United Arab Emirates (UAE), a lack of recognition of community pharmacists as primary HCPs presents a significant barrier to the performance of extended services [69]. Patients and GPs may also consider pharmacy practices to be business-orientated services rather than patient-orientated professional health services, which could hinder the implementation of extended pharmacy services in countries such as Jordan, Japan, Singapore, Hong Kong, Russia and UAE [69].

**Figure 2:**
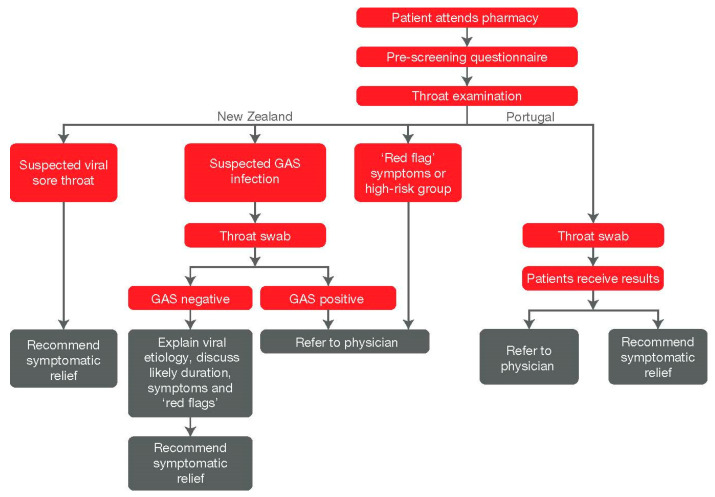
Pharmacy screening process and POC testing intervention in community pharmacies in New Zealand and Portugal. GAS: group A Streptococcus.

There are a number of sore throat POC testing partnerships and initiatives that have been carried out, or that are currently ongoing in community pharmacies in some countries. The New Zealand sore throat screening pilot was a POC testing initiative started in Winter 2019, with the aim of reducing antibiotic use for URTIs [86]. In this multicenter initiative, 559 patients visiting participating community pharmacies were offered a free POC test for GAS pharyngitis. Approximately 96% of patients tested negative for GAS pharyngitis and were offered symptomatic relief. Only 1.6% of patients were referred to a physician following POC testing and patient satisfaction rates were high. A similar project was carried out in pharmacies across Portugal [86]. As part of this initiative, 51 patients underwent a GAS POC test, of whom 17.6% were positive for GAS pharyngitis. Of the patients tested, only 11.1% were referred to a physician following the POC test. The pharmacy screening process and methodology for implementing the POC testing in the New Zealand and Portugal pilot studies is given in Figure 2.

**Figure 3:**
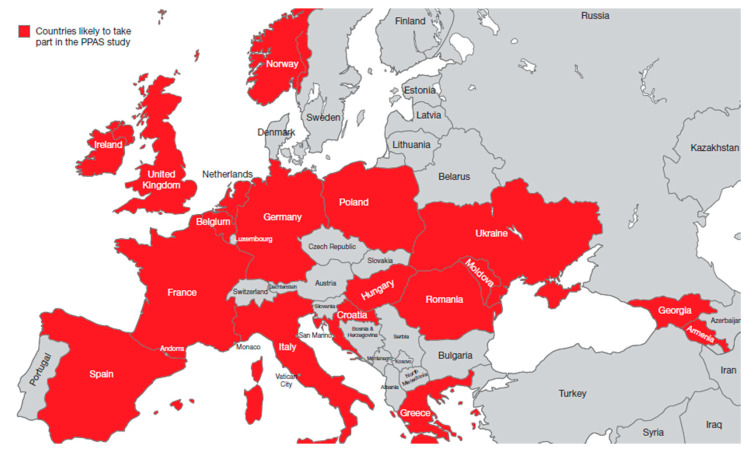
European countries expected to take part in the VALUE-Dx Point Prevalence Audit Survey (PPAS) study.

The VALUE-Dx project in Europe is an ongoing initiative which aims to generate evidence on the value of new diagnostics in appropriate antibiotic prescribing for community-acquired acute respiratory tract infections (CA-aRTI) [87]. The project involves two clinical studies. The first, a point prevalence audit survey (PPAS), will investigate the clinical presentation and management of patients seeking healthcare in approximately 20 European countries (Figure 3) [87]. The second trial, a randomized controlled trial set to be carried out during the winter seasons of 2020–2022, will examine whether POC diagnostics in community care enhance the quality of antibiotic prescribing for CA-aRTI [87].

## 10. Conclusions

Pharyngitis (sore throat) is generally a viral, self-limiting condition which gets better without treatment [6,7,8], although patients will benefit from symptomatic management [54,55]. However, a small proportion of sore throats caused by GAS infection may benefit from the provision of antibiotics [5,20]. Clinical examination and triage without testing for GAS is not able to reliably identify those with infections where GAS is implicated [29,30,31]. The use of GAS POC testing in pharyngitis is a relatively accurate alternative and avoids the 18–48 h delay in results caused by sending traditional throat swabs to the microbiology lab [22,34]. Pharmacists are first-line care providers who are ideally placed to carry out POC testing [56,88] and provide evidence-based symptomatic treatments [53]. Moreover, POC tests are straightforward to administer after some limited training, making them appropriate to be administered in the pharmacy setting. POC testing in the pharmacy can reduce the need for consultation with a physician and can be more cost effective than doctor consultations in healthcare settings in multiple countries [44,48,59,89].

Support and training for pharmacists are essential to enable appropriate use of POC testing for GAS [27,58]. In particular, a good dialogue between pharmacists and GPs will support the process of pharmacists using POC testing [58]. Developing this dialogue is in itself a beneficial effect of POC testing as it may help in other areas of care. Evidence suggests that POC testing in the community pharmacy setting can facilitate appropriate antibiotic use, is an important element of antimicrobial stewardship and could improve public health [16,44,45,46,48,49], which could prove useful in the battle against antibiotic resistance.

POC testing in community pharmacies presents opportunities and challenges for antibiotic stewardship. Its success is contingent on an adequately enforced regulatory system and good interprofessional relationships between doctors and pharmacists.
